# Supplementary material for: Quorum sensing modulates the formation of virulent Legionella persisters within infected cells
Source: Nat Commun. 2019 Nov 18;10:5216. doi: 10.1038/s41467-019-13021-8 (PMC6861284; doi:10.1038/s41467-019-13021-8)
Supplement: Supplementary file 1 — Supplementary Information [file 41467_2019_13021_MOESM1_ESM.pdf]

SUPPLEMENTARY INFORMATION

Quorum sensing modulates the formation of virulent *Legionella* persisters within infected cells

Personnic et al.

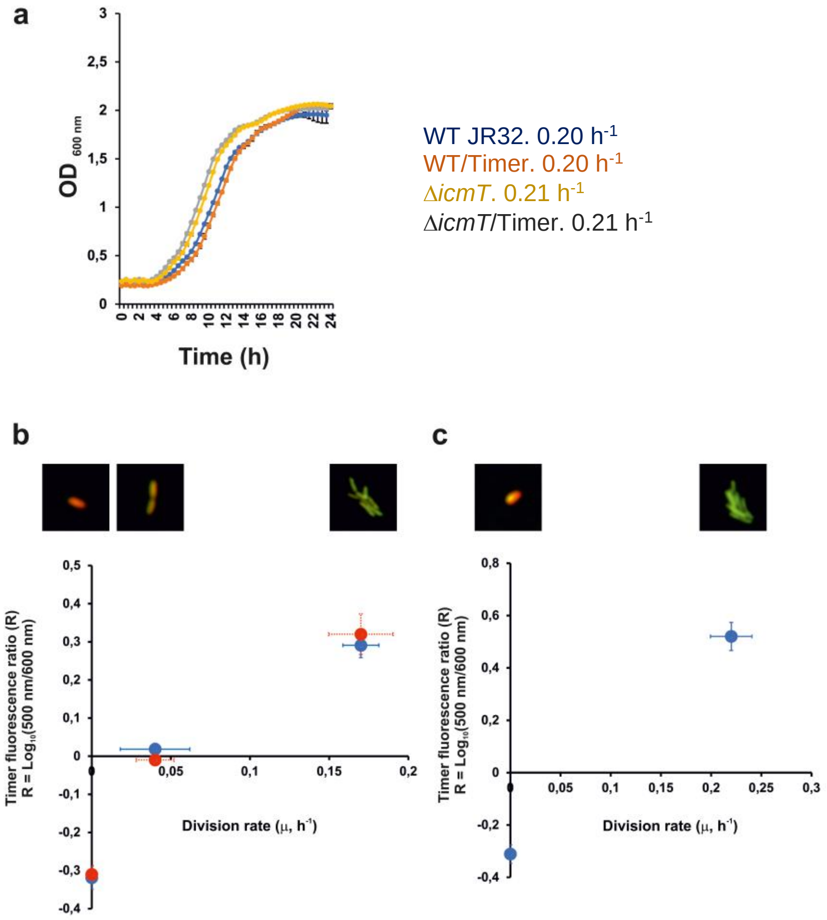

Supplementary Figure 1. Timer fluorescence ratio correlates with bacterial division rate. Related to Figure 1.

(**a**) Timer production does not impact bacterial growth *in vitro*. Growth assays were performed in AYE at 37°C. The calculated exponential growth rates are indicated.

(**b-c**) Timer color ratios correlate with bacterial division rates *in vitro* and during infection. *L. pneumophila*/Timer was immobilized in AYE/0.5 % agarose[35]. Subsequently, colony formation, originating from single bacteria, was monitored by confocal microscopy for (**b**) 16 h at 25°C or (**c**) 12 h at 37°C (plotted in blue, micrographs). Timer color ratios at 500 nm and 600 nm were determined at a single cell level by confocal microscopy and correlated to the number of division that occurred. A similar analysis was performed in infected *A. castellanii* for 24 h (**b**, plotted in red). A minimum of 50 bacteria were analyzed for each experimentally

determined division rate. Data represent the mean ± SEM of three biological replicates (n = 3; light grey filled circles). Source data are provided as a Source Data file.

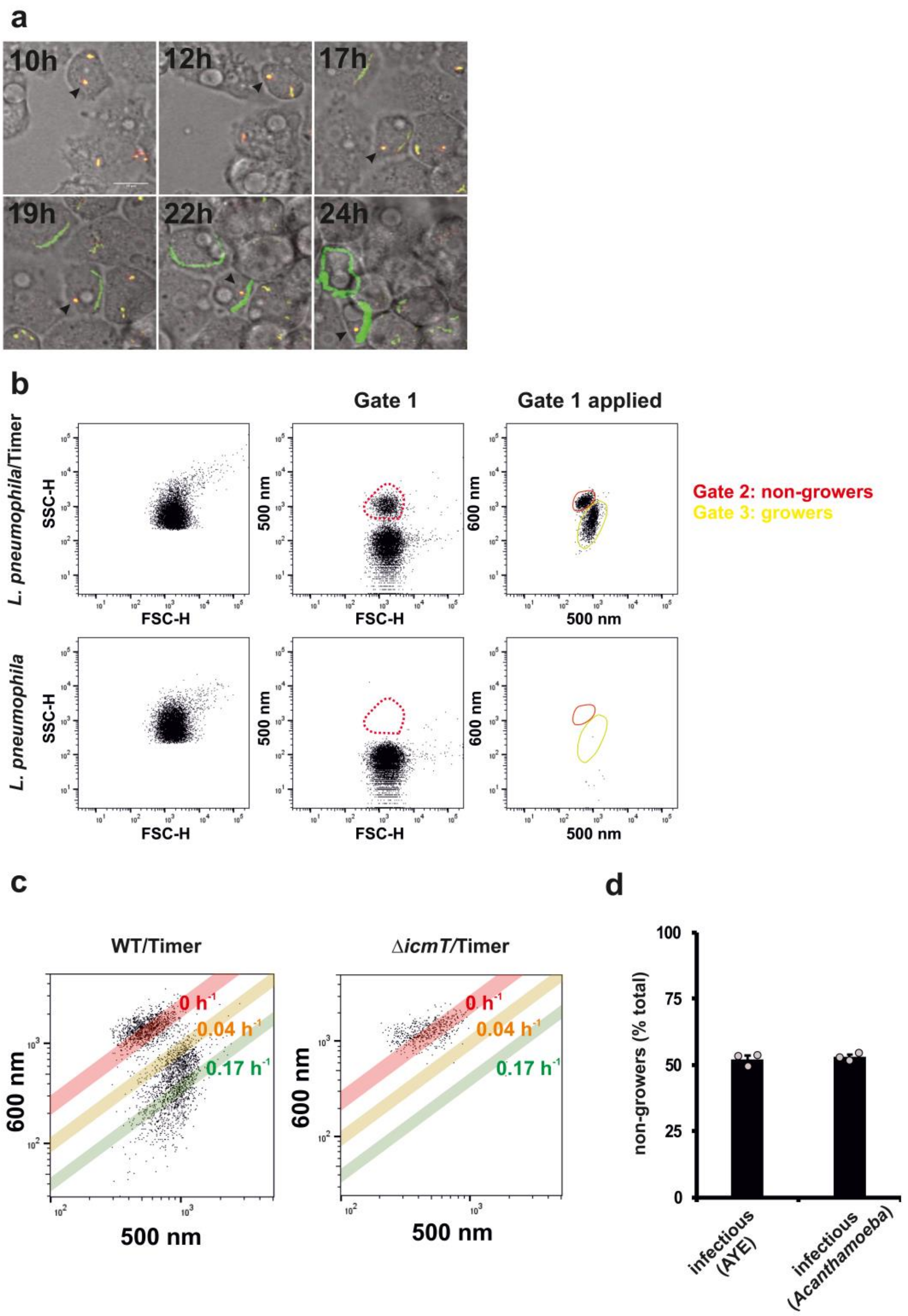

Supplementary Figure 2. Intracellular *L. pneumophila* growth rate heterogeneity in *A. castellanii*. Related to Figure 1b, c, d, e and Figure 2a, b.

(**a**) *A. castellanii* was infected (MOI 1, 24 h) with *L. pneumophila*/Timer, and bacterial replication was monitored by time lapse confocal microscopy. Intracellular growth caused a

color change from red/orange to green, non-growing bacteria remained red/orange (black arrowhead).

(**b**) Flow-cytometry analysis of *L. pneumophila*/Timer in infected cell lysates. *A. castellanii* was infected (MOI 1, 24 h) with *L. pneumophila* producing timer (upper panels) or not (lower panels) and lysed to release intracellular bacteria prior to flow-cytometry analysis. The spectral properties of Timer proteins allow to separate Timer signals from host autofluorescence (gate 1, upper panels). The use of non-fluorescent *L. pneumophila* (lower panels) confirms the absence of any background particles with similar fluorescence. Gate 2 and Gate 3 correspond to the non-growers and the growers, respectively.

(**c**) Identification of the intracellular *L .pneumophila* subpopulations. *A. castellanii* was infected (MOI 1, 24 h) with *L. pneumophila* WT and Δ*icmT* producing Timer, lysed and the released bacteria were analyzed by flow-cytometry. Experimentally determined Timer color ratios for *L. pneumophila in vitro* growth and the related division rates are shown (red, non-growers, NG, $\mu = 0\ h^{-1}$; yellow, slow-growers, G_S, $\mu \approx 0.04\ h^{-1}$; green, fast-growers, G_F, $\geq 0.17\ h^{-1}$).

(**d**) Infectious *L. pneumophila* generates intracellular non-growers upon infection. *A. castellanii* was infected (MOI 1, 24 h) either with stationary phase *L. pneumophila* grown in AYE broth, or with bacteria obtained from natural lysis of *A. castellanii* infected with *L. pneumophila* in COMBO freshwater medium. Subsequently, the infected amoebae were lysed, and the fraction of non-growers was determined by flow cytometry.

Data represent the mean ± SEM of three biological replicates (*n* = 3; light grey filled circles). Source data are provided as a Source Data file.

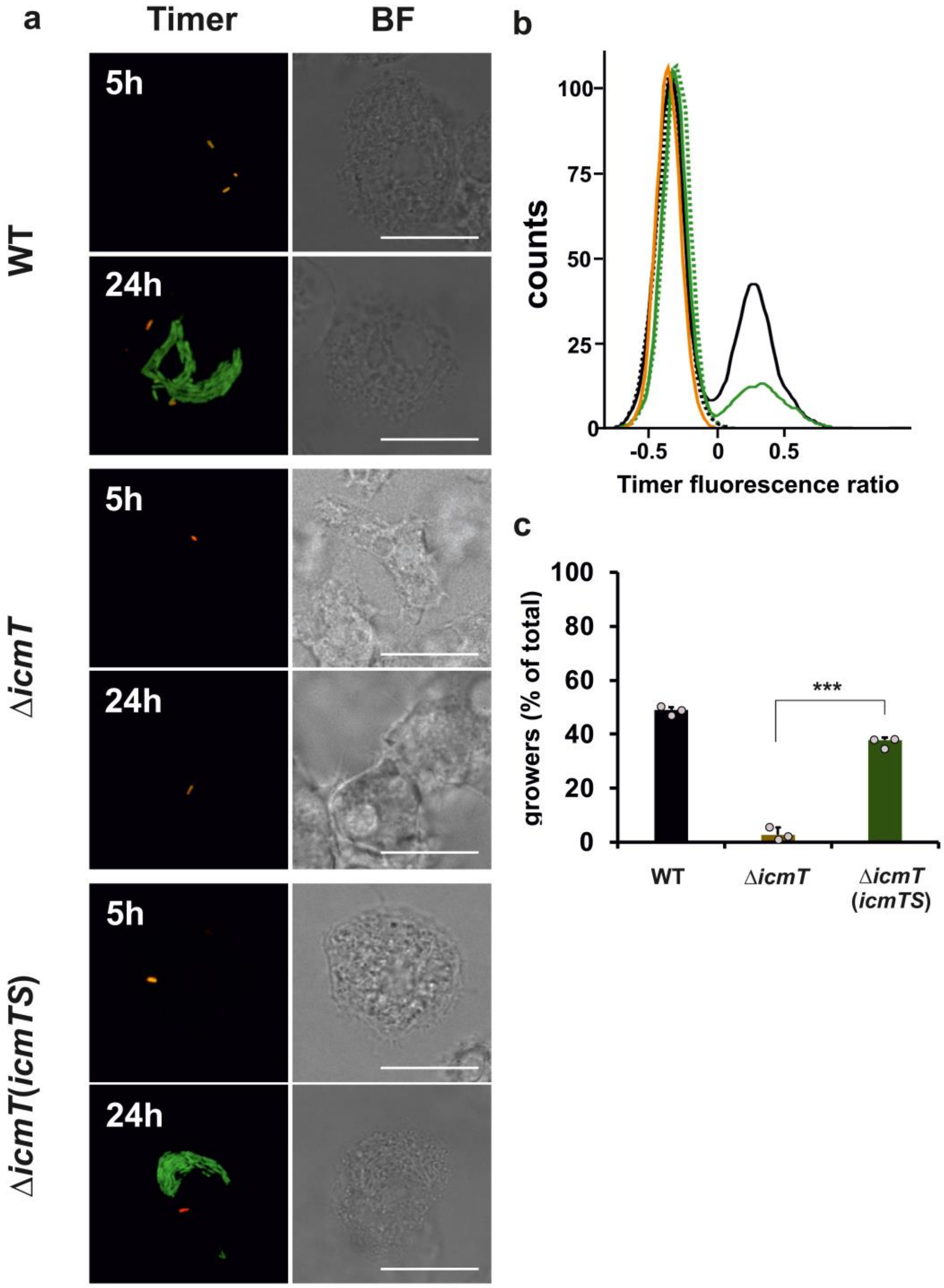

Supplementary Figure 3. Complementation of *L. pneumophila* Δ*icmT*/Timer growth phenotype in *A. castellanii*. Related to Figure 1b, c, d, e and Figure 4b. *A. castellanii* was infected (MOI 1) with *L. pneumophila* WT, the avirulent Δ*icmT* strain or the complemented strain Δ*icmT*(*icmTS*), producing Timer. Intracellular growers and non-growers were detected by (**a**) confocal microscopy 5 h and 24 h p.i. or (**b**) by flow cytometry (24 h p.i.). Micrographs

show the overlay of Timer fluorescence (500 nm and 600 nm) and bright field. Scale bar, 20 μm. Black, WT; orange, Δ*icmT*; dashed green, Δ*icmT*(*icmTS*). (**c**) The size of the growing subpopulation was determined by flow cytometry for each strain (24 h p.i.).

Data represent the mean ± SEM of three biological replicates (n = 3; light grey filled circles). Student's t test two-tailed. *** $P < 0.001$. Source data are provided as a Source Data file.

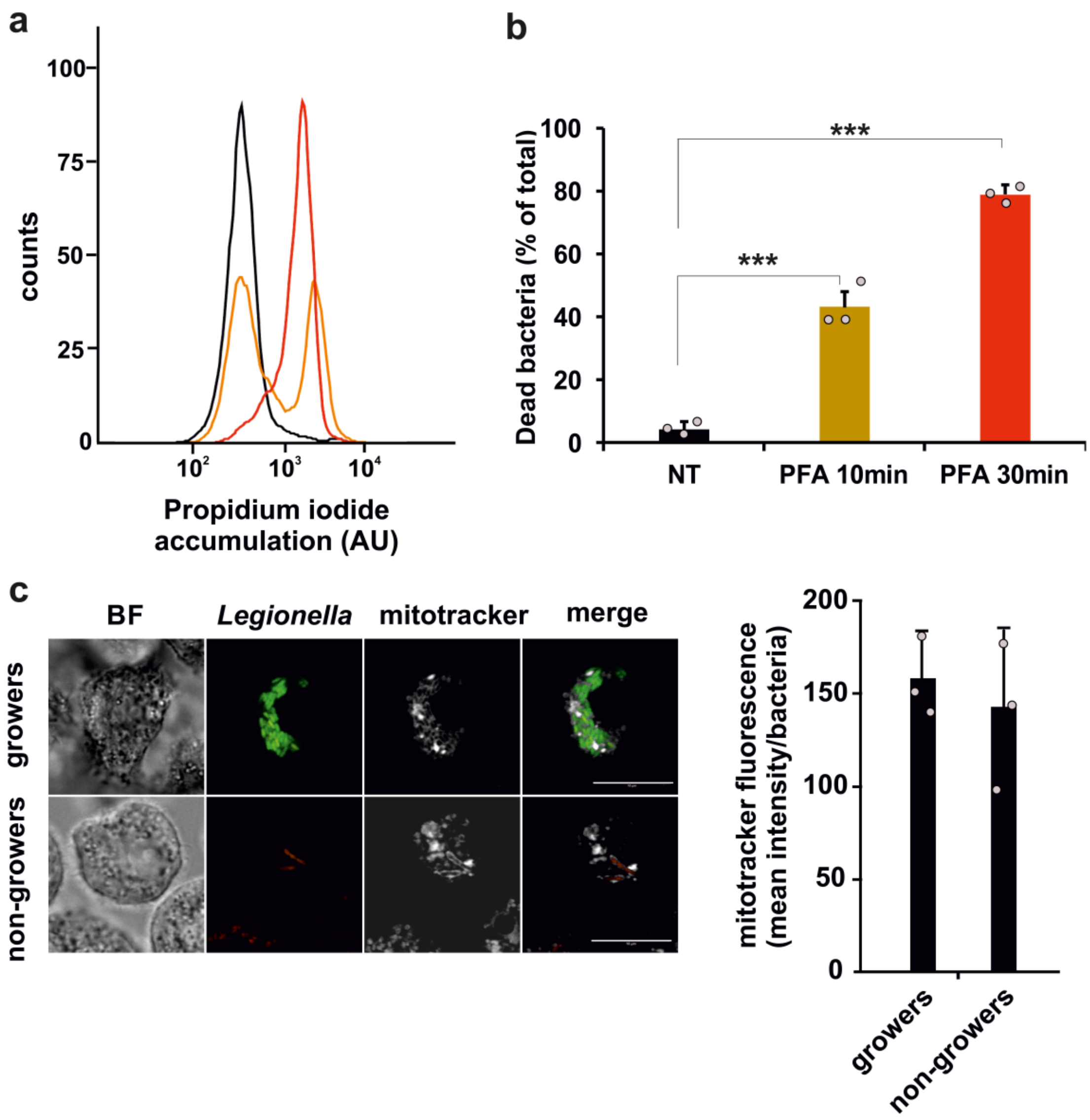

Supplementary Figure 4. Intracellular *L. pneumophila* are viable. Related to Figure 1f and Figure 2c.

(**a-b**) *A. castellanii* was infected (MOI 1, 24 h) with *L. pneumophila*/GFP. Lysates of infected cells were subsequently incubated with propidium iodide (PI; 30 min, 10 μg.mL^{-1}), and bacterial viability was determined by flow cytometry by gating on GFP positive bacteria. To kill bacteria while preserving bacterial GFP fluorescence, lysates were PFA-treated prior to PI-staining. Representative frequency graph (**a**) and quantification (**b**). Black, non-treated (NT); yellow, PFA 10 min; red; PFA, 30 min.

(**c**) Intracellular non-growers maintain a membrane potential. *A. castellanii* were infected (MOI 1, 24 h) with *L. pneumophila*/Timer, stained with MitoTracker-DeepRed (indicating membrane potential) and analyzed by confocal microscopy (100 growing and non-growing bacteria each). BF, bright field; scale bar, 10 μm.

Data represent the mean ± SEM of three biological replicates ($n = 3$; light grey filled circles). Student's t test two-tailed. *** $P < 0.001$. Source data are provided as a Source Data file.

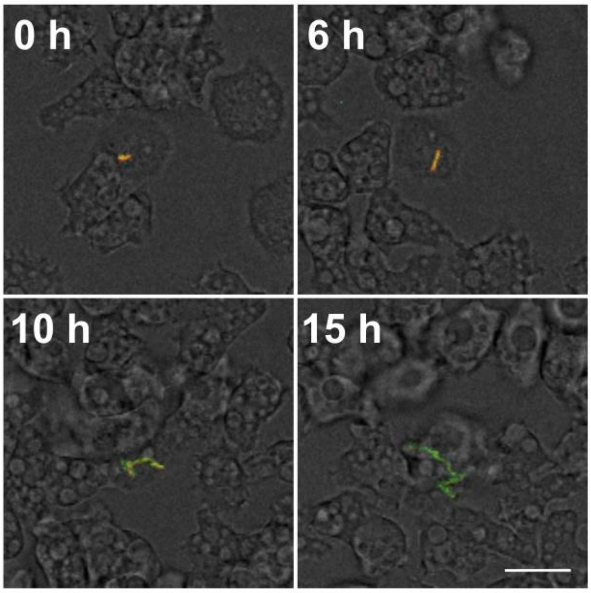

Supplementary Figure 5. FACS-sorted *L. pneumophila* non-growers resume growth upon reinfection. Related to Figure 1f, Figure 2c and Supplementary Figure 4. *A. castellanii* was infected (MOI 1, 24 h) with *L. pneumophila*/Timer. Subsequently, FACS-sorted *L. pneumophila* non-growers were used to infect fresh *A. castellanii*, and bacterial growth resumption was monitored by time lapse confocal microscopy. Intracellular growth caused a color change from red/orange to green. Time p.i. is indicated in the upper left corner. Micrographs show the overlay of bright field and the Timer fluorescence at 500 nm and 600 nm. Scale bar, 20 μm.

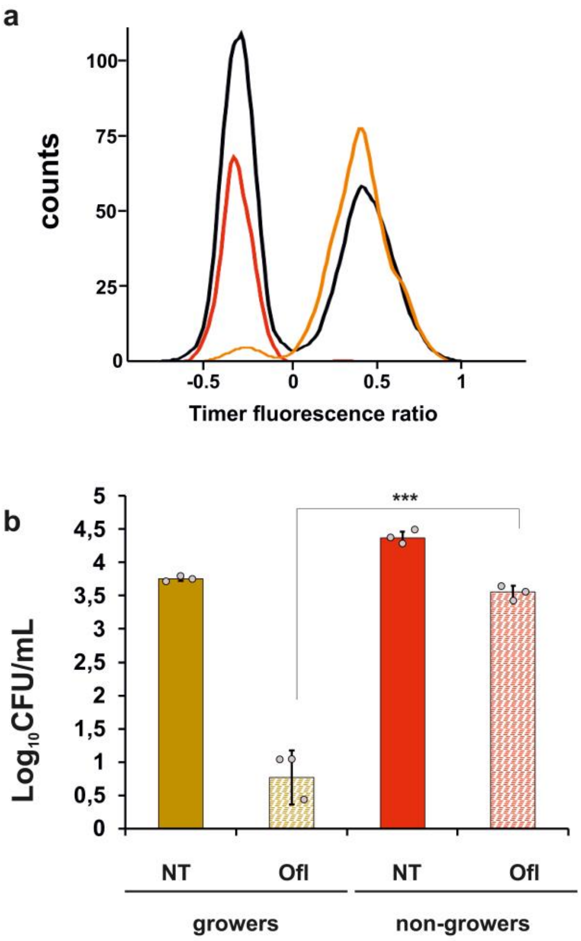

Supplementary Figure 6. Increased antibiotics tolerance of intracellular non-growers *in vivo*. Related to Fig. 2e and f. *A. castellanii* was infected with *L. pneumophila*/Timer (MOI 1, 24 h), and treated or not with a high concentration of ofloxacin (300 μg.mL^{-1}, 1 h). Subsequently, 10^5 growing and 10^5 non-growing *L. pneumophila* were FACS-sorted for each condition and plated to evaluate antibiotic susceptibility of the intracellular subpopulations. (**a**) Subpopulations sorted after ofloxacin treatment were re-analyzed by flow cytometry and compared to the initial infected cell lysate (pre-sort) to evaluate the separation efficiency. Pre-sort, black; non-growers, red; growers, yellow. (**b**) *L. pneumophila* subpopulation survival. NT, non-treated; Ofl, ofloxacin treated cells.

Data represent the mean ± SEM of three biological replicates (n = 3; light grey filled circles). Student's t test two-tailed. *** $P < 0.001$. Source data are provided as a Source Data file.

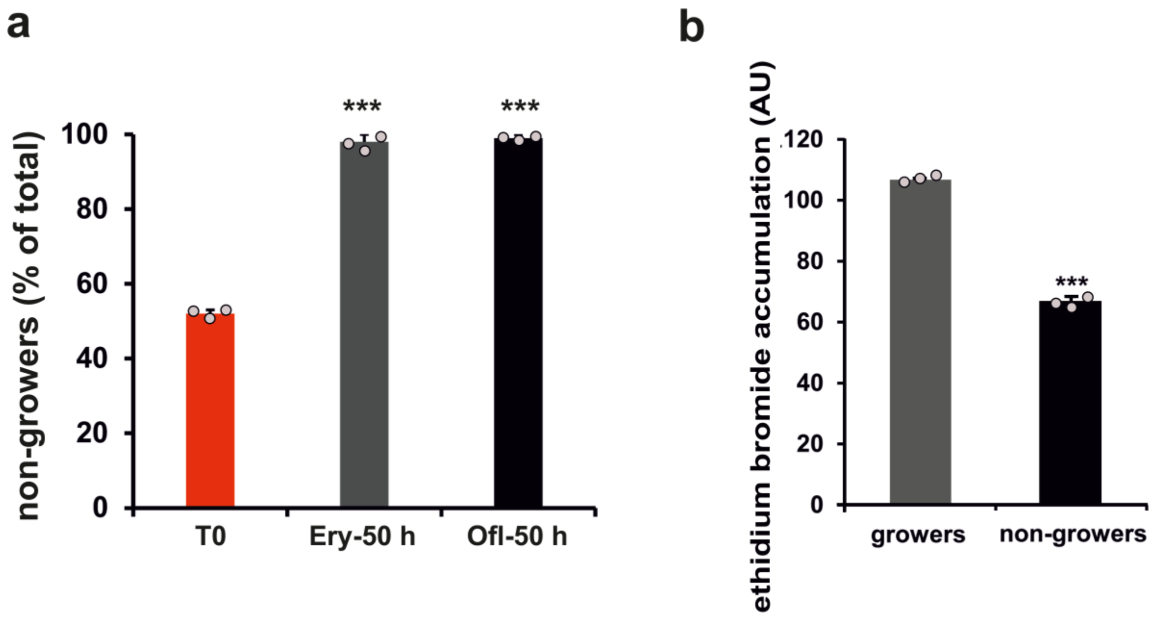

Supplementary Figure 7. Formation of *L. pneumophila* persisters in infected amoebae. Related to Figure 2e, f and Supplementary Figure 6. *A. castellanii* was infected (MOI 1, 24 h) with *L. pneumophila*/Timer.

(**a**) Antibiotic exposure produces a homogeneous population of non-growing bacteria. After host cell lysis, bacteria were resuspended in AYE supplemented with antibiotics. After 50 h of antibiotic treatment, bacteria were plated (figure 2f) or analyzed by flow cytometry and the fraction of non-growers was determined by flow cytometry. T0, before treatment; Ery, erythromycin (60 μg.mL^{-1}); Ofl, ofloxacin (30 μg.mL^{-1}). Only non-growers survive the antibiotic treatment.

(**b**) Intracellular non-growers show high drug efflux activity. FACS-sorted growing and non-growing *L. pneumophila* were treated with ethidium bromide (1 μg.mL^{-1}, 1 h), and dye accumulation was quantified by flow cytometry for each subpopulation.

Data represent the mean ± SEM of three biological replicates ($n = 3$; light grey filled circles). Student's t test two-tailed. *** $P < 0.001$. Source data are provided as a Source Data file.

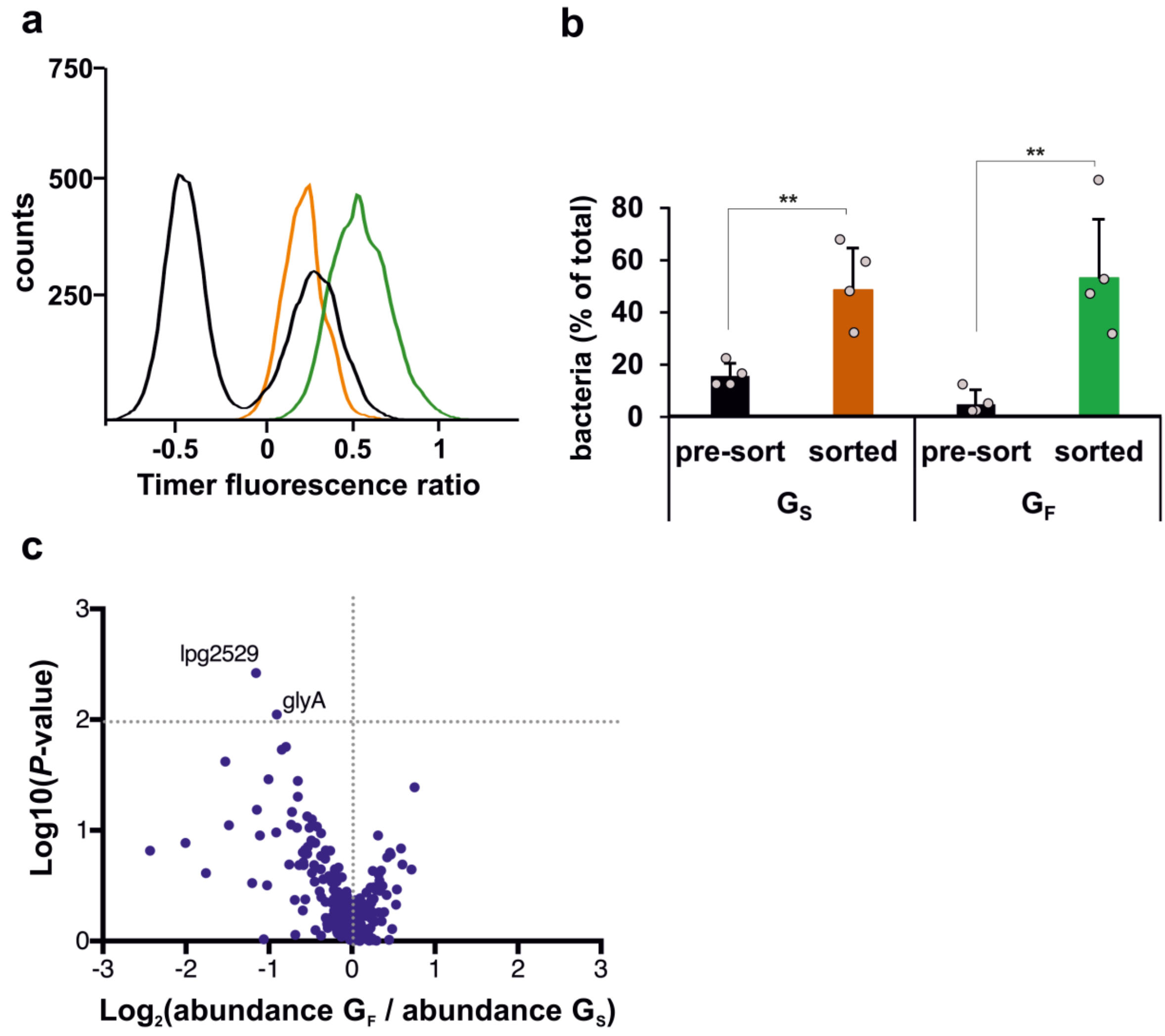

Supplementary Figure 8. Proteome comparison of intracellular slow- and fast-growing subpopulations. Related to Figure 3.

(**a**, **b**) FACS-sorting of intracellular *L. pneumophila* subpopulations. *A. castellanii* was infected with *L. pneumophila*/Timer (MOI 1, 24 h) and lysed. Released intracellular bacteria were FACS-sorted according to their Timer green/red color ratio, sorted subpopulations were re-analyzed by flow cytometry and compared to the initial infected cell lysate (pre-sort) to evaluate the separation efficiency between fast-growers (G_F) and slow-growers (G_S). (**a**) Graph and (**b**) quantification is shown. Pre-sort, black; G_S, slow-growers, orange; G_F, fast-growers, green.

(**c**) Comparative proteomics of sorted fast-growers (G_F) and slow-growers (G_S) shows very similar signatures. Protein abundance in each subpopulation is depicted as volcano plot (see Supplementary Data 2 for the full data set).

Data represent the mean ± SEM of four biological replicates (*n* = 4; light grey filled circles). Student's t test two-tailed; ** $P < 0.01$. Source data are provided as a Source Data file.

a

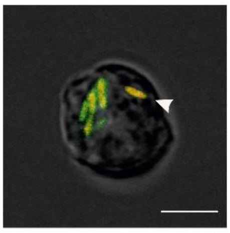

b

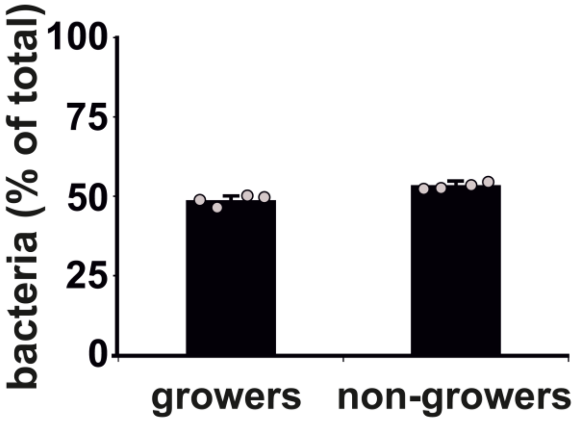

Supplementary Figure 9. Intracellular *L. pneumophila* growth rate heterogeneity in *D. discoideum*. Related to Figure 4c and Figure 5f. *D. discoideum* amoebae were infected (MOI 1, 24 h) with *L. pneumophila*/Timer, and intracellular bacterial growth rate heterogeneity was evaluated by (**a**) confocal microscopy (intact cells) and (**b**) flow cytometry (cell lysates). The micrograph shows an overlay of bright field images and Timer fluorescence at 500 nm and 600 nm. White arrowhead: intracellular non-growing bacterium. Scale bar, 10 μm. Histograms show the fractions of intracellular growers and non-growers.

Data represent the mean ± SEM of four biological replicates (*n* = 4; light grey filled circles). Source data are provided as a Source Data file.

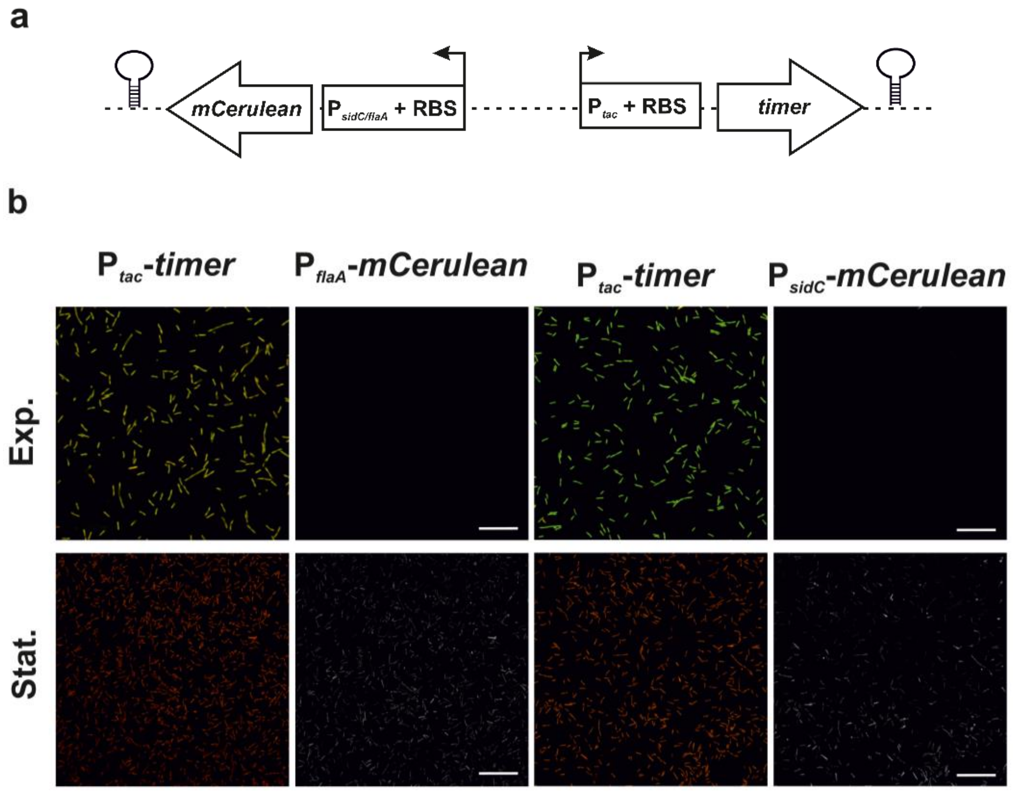

Supplementary Figure 10. Dual fluorescence reporter for *L. pneumophila* growth rate and gene expression. Related to Figure 5a, b, and f, and Supplementary Figure 15.

(**a**) Genetic organization of the fluorescence reporter for both growth rate and gene expression. The fluorescence reporter construct comprises *timer* under the control of the P_{tac} promoter, rendered constitutive, and an optimized ribosome binding site (RBS) for protein production. The expression of *mCerulean* is controlled by the promoter and RBS of hallmark genes for motility (P_{flaA}) or virulence (P_{sidC}). A hairpin transcriptional terminator is inserted downstream of *timer and mCeruelan*.

(**b**) Production of mCerulean by *L. pneumophila* (P_{tac}-*timer* - $P_{flaA/sidC}$-*mCerulean*) depends on the growth phase. *L. pneumophila* (P_{tac}-*timer* - $P_{flaA/sidC}$-*mCerulean*) was grown in AYE broth to exponential (Exp.) and stationary phase (Stat.) and analyzed by confocal microscopy. Micrographs show the fluorescence for Timer at 500 nm and 600 nm, and for mCerulean at 479 nm. Stationary phase grown bacteria appear red/orange and produce mCerulean, in agreement with being growth arrested and motile/virulent (transmissive). Scale bar, 20 μm.

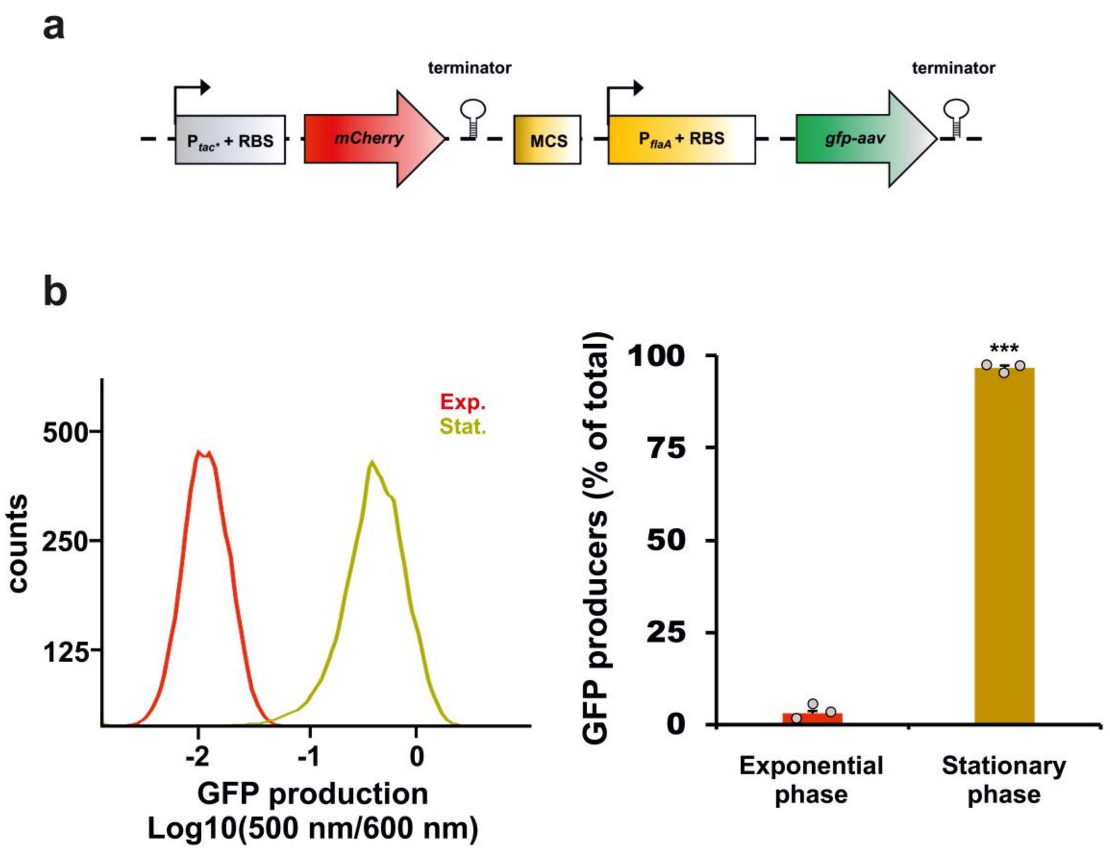

Supplementary Figure 11. Detection of transmissive *L. pneumophila* at single cell level. Related to Figure 5c, d, e, Figure 6 c, d, e, and Figure 7e

(**a**) Genetic organization of the dual fluorescence reporter. The dual fluorescence reporter construct comprises *mCherry* under the control of the P_{tac} promoter, rendered constitutive, and an optimized ribosome binding site (RBS) for protein production. The expression of *gfp* is controlled by a promoter and RBS of interest. Downstream of *mCherry*, a hairpin transcriptional terminator suppresses potential polar effect on the expression of *gfp*. The *flagellin* promoter and RBS to control *gfp* expression are inserted into a multiple cloning site (MCS). The GFP degradation rate is increased by adding the C-terminal peptide tag AANDENYAAAV, allowing to quantify changes in promoter activity by the fluorescence intensity ratio [500 nm (GFP)/600 nm (mCherry)].

(**b**) Production of GFP by *L. pneumophila* (P_{flaA}-*gfp*) depends on the growth phase. *L. pneumophila* (P_{flaA}-*gfp*) were grown in AYE broth to exponential (Exp., red) and stationary

phase (Stat., yellow) and analyzed by flow cytometry (left panel). The color ratio – Log_{10}[500 nm (green)/600 nm (red)] – was calculated for each individual bacterium and plotted in frequency graphs (right panel).

Data represent the mean ± SEM of three biological replicates ($n = 3$; light grey filled circles). Student's t test two-tailed; *** $P < 0.001$. Source data are provided as a Source Data file.

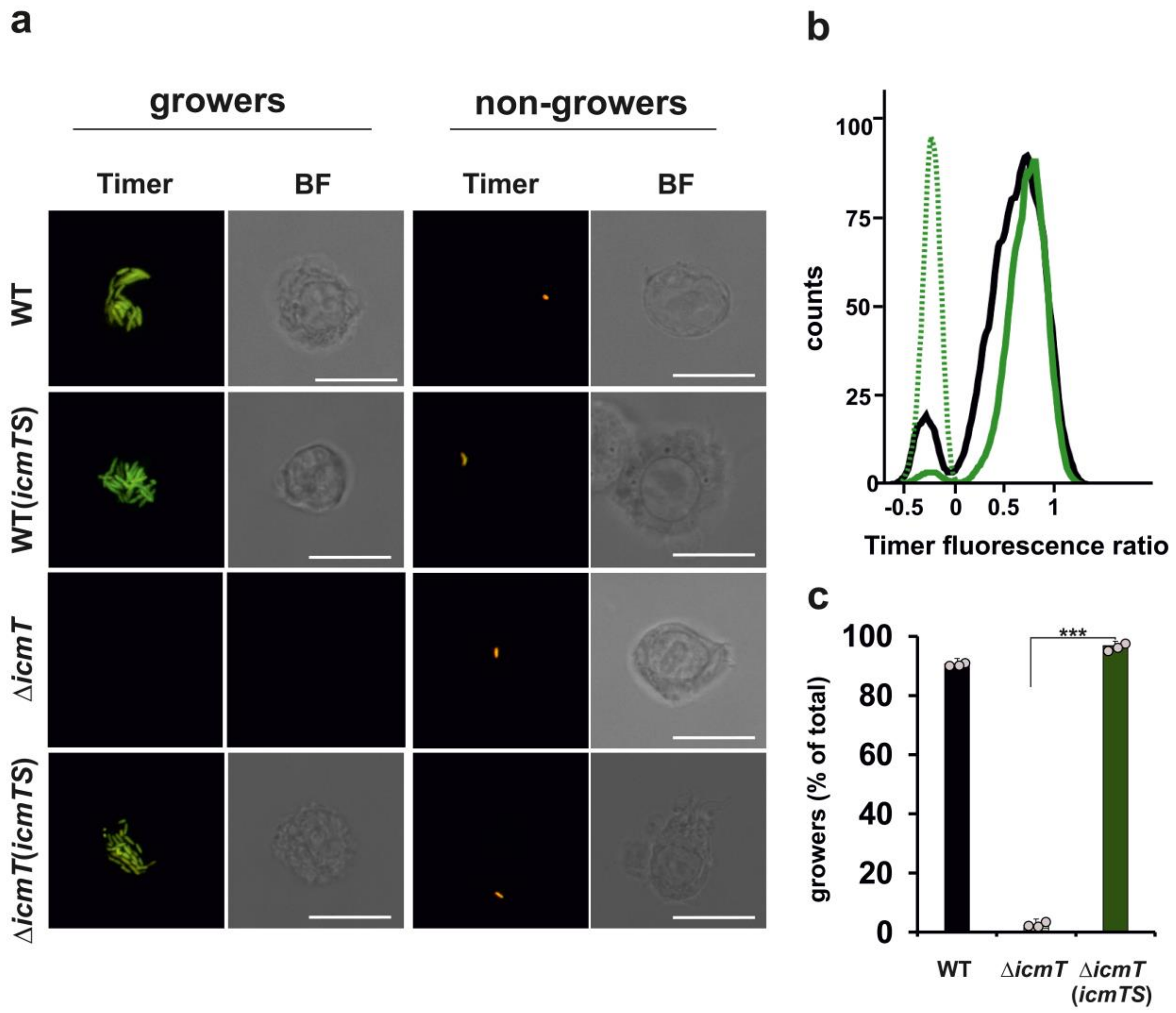

Supplementary Figure 12. Complementation of *L. pneumophila* Δ*icmT*/Timer growth phenotype in murine macrophages. Related to Figure 6a and b. Murine macrophages were infected (MOI 1, 24 h) with *L. pneumophila* WT, WT(*icmTS*), the avirulent Δ*icmT* strain and the complemented strain Δ*icmT*(*icmTS*), producing Timer. Intracellular growers and non-growers were detected by (**a**) confocal microscopy (micrographs show Timer fluorescence (500 nm / 600 nm) and bright field. Scale bar, 20 μm) or (**b**) flow cytometry (WT, black); Δ*icmT* (dashed green); Δ*icmT*(*icmTS*) (green) in the cell lysates. (**c**) The size of the growing subpopulation was determined by flow cytometry analysis of the infected cell lysates.

Data represent the mean ± SEM of three biological replicates (*n* = 3; light grey filled circles). Student's t test two-tailed. *** $P < 0.001$. Source data are provided as a Source Data file.

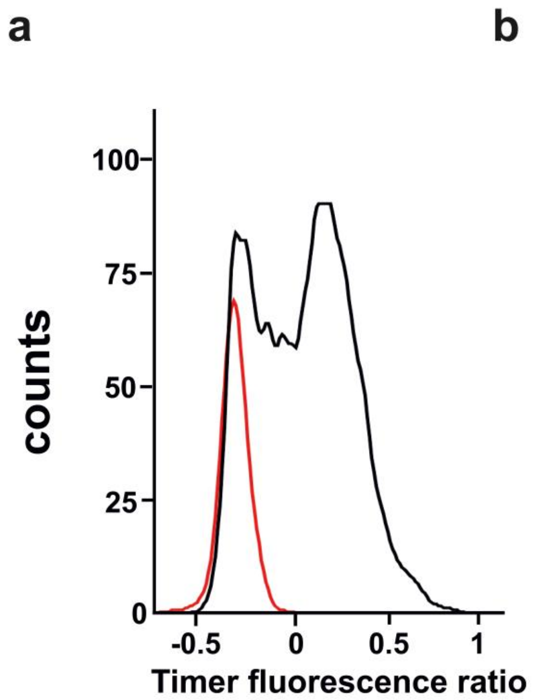

Supplementary Figure 13. FACS-sorted *L. pneumophila* non-growers produced in IFN-γ treated macrophages resume growth upon reinfection. Related to Figure 6a and b. IFN-γ treated murine macrophages were infected (MOI 1, 24 h) with *L. pneumophila*/Timer. After host cell lysis, FACS-sorted non-growers were used to infect *A. castellanii*. (**a)** FACS-sorted non-growers (red) were re-analyzed by flow cytometry and compared to the initial infected cell lysate (pre-sort, black) to evaluate the separation efficiency. (**b**) Bacterial growth resumption was monitored by confocal microscopy. Intracellular growth caused a color change from red/orange to green. Micrographs show the Timer fluorescence at 500 nm and 600 nm as well as the bright field. Time p.i. is indicated in the upper left corner. Scale bar, 20 µm.

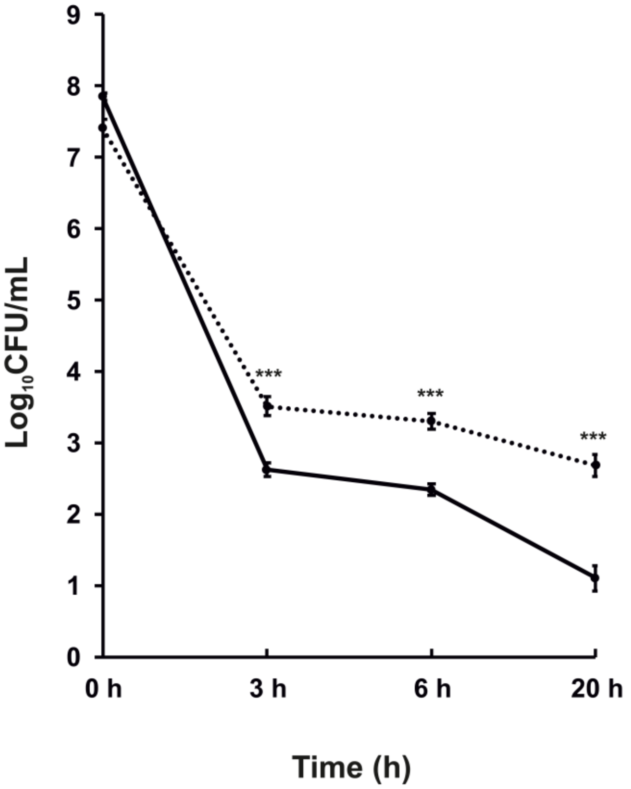

Supplementary Figure 14. ***L pneumophila*** **persistence to antibiotic exposure is enhanced in IFNγ-treated macrophages.** Related to Figure 6a and b. Naïve and IFN-γ-treated murine macrophages were infected (MOI 1, 24 h) with *L. pneumophila*/Timer. After host cell lysis, bacteria were resuspended in AYE supplemented with ofloxacin (30 μg.mL^{-1}), incubated at 37°C and plated at indicated time-points. Bacteria extracted from naïve macrophages (black), bacteria extracted from IFNγ-treated macrophages (dashed). Bi-phasic kill curve reveals presence of persisters.

Data represent the mean ± SEM of three biological replicates (n = 3; light grey filled circles). Student's t test two-tailed, *** P < 0.001; naïve vs. IFN-γ-treated macrophages). Source data are provided as a Source Data file.

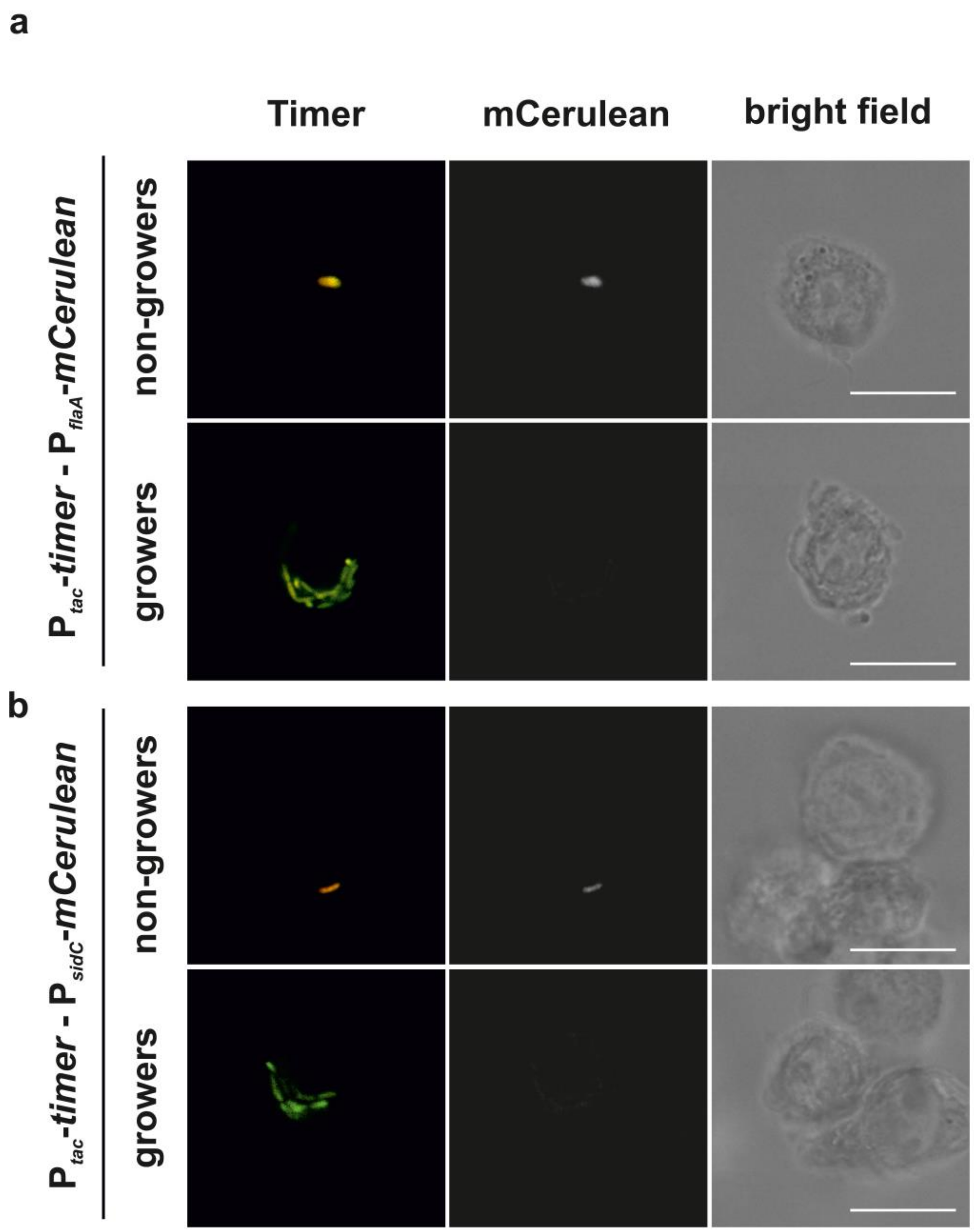

Supplementary Figure 15. Intracellular *L. pneumophila* non-growers express hallmark genes of motility and virulence during macrophage infection. Related to Figure 6c, d and e. Murine macrophages were infected (MOI 1, 24 h) with *L. pneumophila* harboring the fluorescent reporters (**a**) P$_{tac}$-*timer* - P$_{flaA}$-*mCerulean* or (**b**) P$_{tac}$-*timer* - P$_{sidC}$-*mCerulean*, and analyzed by confocal microscopy. Micrographs show the fluorescence for Timer at 500 nm and 600 nm, for mCerulean at 479 nm and the bright field. Intracellular non-replicating bacteria appeared red/orange and produced mCerulean. Scale bar 20 μm.

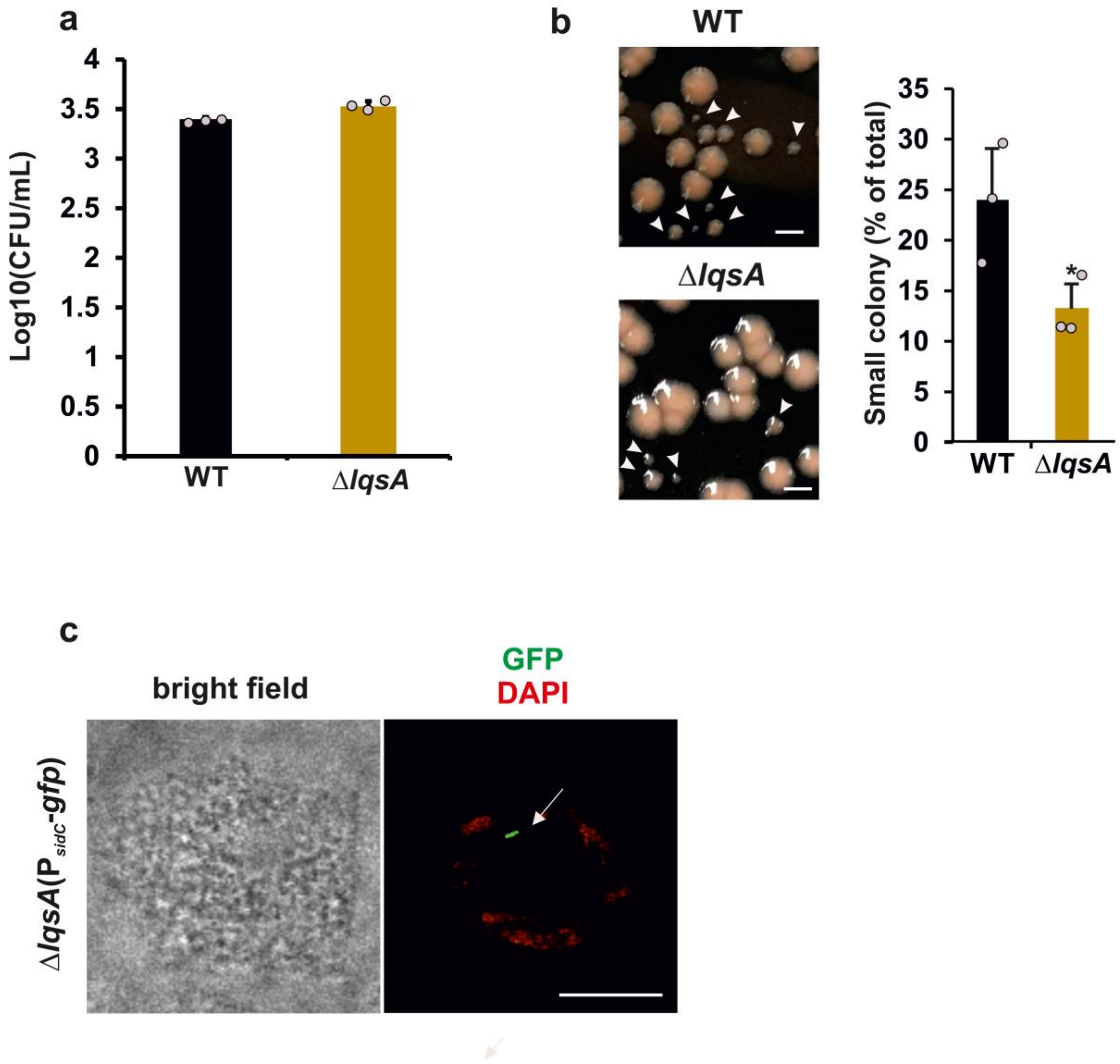

Supplementary Figure 16. LqsA promotes the formation of intracellular virulent non-replicating persisters. Related to Figure 7a, b, c, d, e.

(**a**) Deletion of *lqsA* does not impair the virulence of *L. pneumophila*/Timer. *A. castellanii* were infected (MOI 1, 24 h) with Timer-producing *L. pneumophila* WT or Δ*lqsA*, lysed and CFU were determined.

(**b**) *lqsA* inactivation reduces the formation of small colonies on CYE agar plates. Lysates of *A. castellanii* infected (MOI 1, 24 h) with Timer-producing *L. pneumophila* WT or Δ*lqsA* were plated on CYE agar, and the formation of colonies was determined. The number of small

colonies (colonies at least ca. 2× fold smaller; white arrowheads) was determined and found to be reduced by ca. 50% for the Δ*lqsA* mutant strain.

(**c**) Production of GFP by intracellular Δ*lqsA*(P_{sidC}-*gfp*) non-growers. *A. castellanii* was infected (MOI 1, 24 h) with Δ*lqsA*(P_{sidC}-*gfp*), fixed and analyzed by confocal microscopy. Micrographs show bright field and fluorescence at 500 nm (GFP, green)/405 nm (DAPI, red). The white arrow indicates an intracellular non-replicating, GFP producing bacterium. Scale bar 20 μm.

Data represent the mean ± SEM of three biological replicates ($n = 3$; light grey filled circles). Student's t test two-tailed; * $P < 0.05$. Source data are provided as a Source Data file.

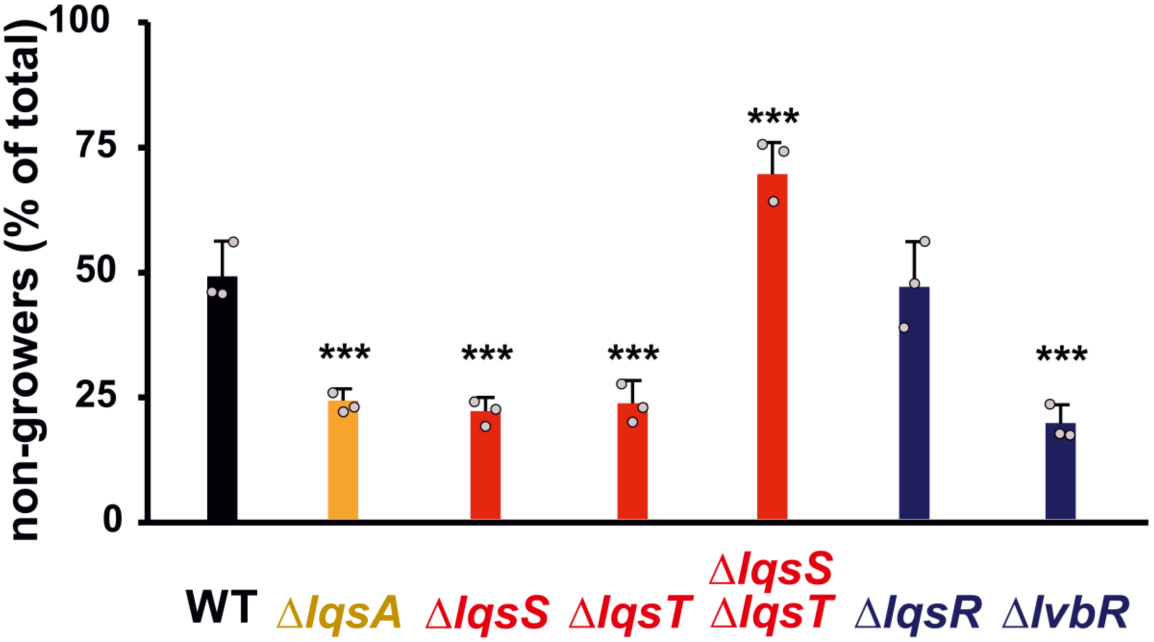

Supplementary Figure 17. The Lqs quorum sensing system controls the formation of intracellular non-growers. Related to Figure 7f. The Lqs quorum sensing system controls the formation of intracellular non-growers. *A. castellanii* was infected (MOI 1, 24 h) with *L. pneumophila* WT or the isogenic Δ*lqsA*, Δ*lqsS*, Δ*lqsT*, Δ*lqsS*Δ*lqsT*, Δ*lqsR* or Δ*lvbR* mutant strains expressing timer, lysed and the fraction of non-growers was determined by flow cytometry. Data represent the mean ± SEM of three biological replicates (n = 3; light grey filled circles). Student's t test two-tailed; *** $P < 0.001$. Source data are provided as a Source Data file.

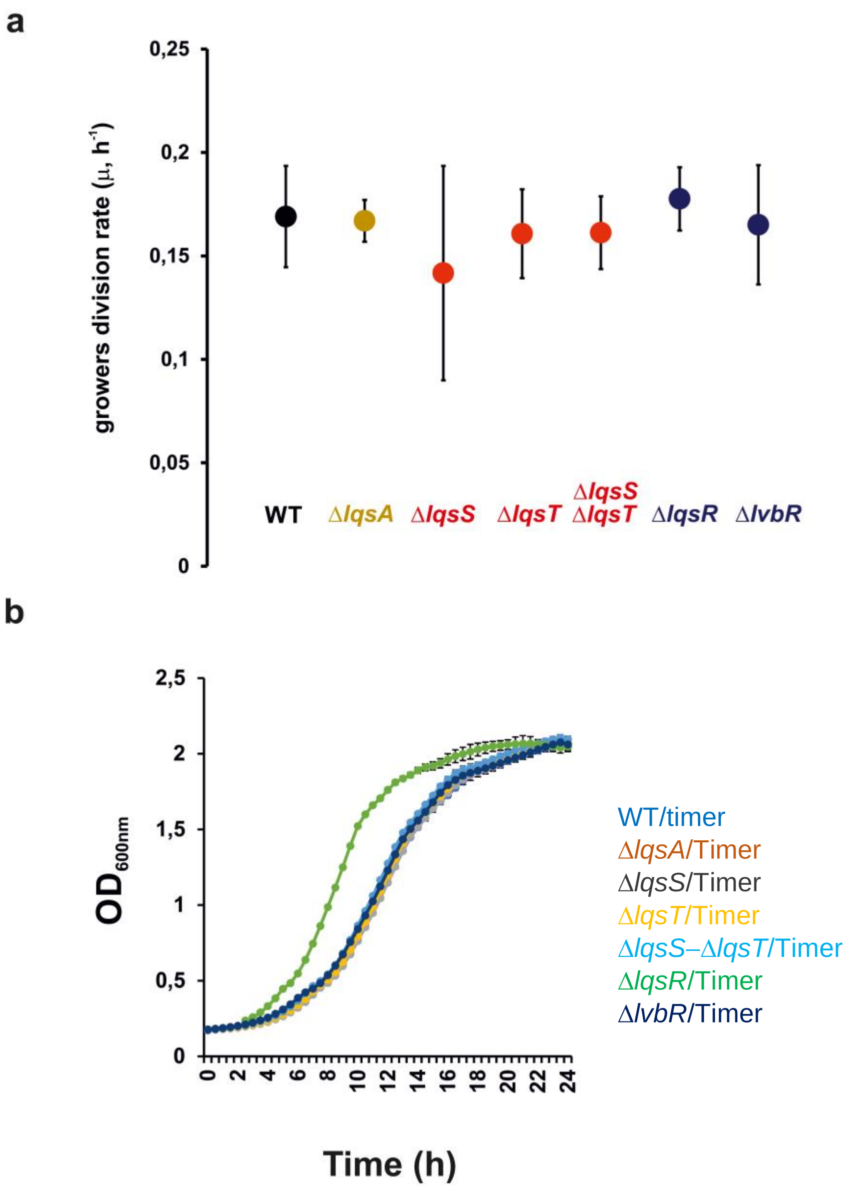

Supplementary Figure 18. Growth rate of the *L. pneumophila* isogenic Δ*lqs* mutants producing Timer. Related to Figure 7. (**a**) Intracellular growth in amoebae. *A. castellanii* was infected (MOI 1, 24 h) with *L. pneumophila* WT, the isogenic Δ*lqsS*, Δ*lqsT*, Δ*lqsS*-Δ*lqsT*, Δ*lqsR* or Δ*lvbR* mutant strains expressing *timer*. After host cell lysis, the median Timer color ratio (R) for the growing subpopulation was determined by flow cytometry to estimate the division rate (μ) using the empirical formula [$\mu = \frac{0.1+\mathrm{Log10R}}{2.6}$], (Supplementary Fig. 1b and c). (**b**) Growth in AYE at 37°C. For WT and all tested mutant strains, the calculated exponential

division rate was 0.19 h^{-1}. Data represent the mean ± SEM of three biological replicates (n = 3; light grey filled circles). Source data are provided as a Source Data file.
