# Supplementary material for: Quorum sensing modulates the formation of virulent Legionella persisters within infected cells
Source: Nat Commun. 2019 Nov 18;10:5216. doi: 10.1038/s41467-019-13021-8 (PMC6861284; doi:10.1038/s41467-019-13021-8)
Supplement: Supplementary file 3 — Description of Additional Supplementary Files [file 41467_2019_13021_MOESM3_ESM.pdf]

Description of Additional Supplementary Files

File Name: Supplementary Data 1

Description: Proteome comparison of intracellular growing and non-growing *L. pneumophila*.
Related to Figure 3. Abundance ratios of proteins in intracellular growing and non-growing subpopulations of FACS-sorted *L. pneumophila*/Timer 24 h p.i. Four biological replicates were pooled into two to increase the number of peptides detected. Statistical difference to a ratio of 1 was tested by two-tailed Student's t test on log-transformed data.

File Name: Supplementary Data 2

Description: Proteome comparison of intracellular slow- and fast-growing *L. pneumophila*.
Related to Figure 3 and Supplementary Figure 8. Abundance ratios of proteins in intracellular slow-growing (GS) and fast-growing (GF) subpopulations of FACS-sorted *L. pneumophila*/Timer 24 h p.i..Four biological replicates were processed. Statistical difference to a ratio of 1 was tested by two-tailed Student's t test on log-transformed data.

File Name: Supplementary Data 3

Description: Cells and strains used in this study.

File Name: Supplementary Data 4

Description: Plasmids used in this study.

File Name: Supplementary Data 5

Description: Oligonucleotides and synthesized gene used in this study.

File Name: Supplementary Movie 1

Description: Some *L. pneumophila* individuals do not resume growth in *A. castellanii*.
Related to Figure 1. *A. castellanii* was infected (MOI 1, 24 h) with *L. pneumophila*/Timer, and bacterial replication was monitored by time lapse confocal microscopy. Intracellular growth caused a color change from red/orange to green, non-growing bacteria remained red. Time p.i. is indicated in the upper left corner.

File Name: Supplementary Movie 2

Description: FACS-Sorted *L. pneumophila* non-growers resume growth in *A. castellanii*.
Related to Figure 1f, Figure 2c and Supplementary Figure 4 and 5. *A. castellanii* was infected (MOI 1, 24 h) with L. pneumophila/Timer. Subsequently, FACS sorted L. pneumophila non-growers were used to infect fresh A. castellanii, and bacterial growth resumption was monitored by time lapse confocal microscopy. Intracellular growth caused a color change from red/orange to green. Time p.i. is indicated in the upper left corner.
