# Supplementary material for: Quorum sensing modulates the formation of virulent Legionella persisters within infected cells
Source: Nat Commun. 2019 Nov 18;10:5216. doi: 10.1038/s41467-019-13021-8 (PMC6861284; doi:10.1038/s41467-019-13021-8)
Supplement: Supplementary file 11 — Reporting Summary [file 41467_2019_13021_MOESM11_ESM.pdf]

nature research

Corresponding author(s): Nicolas Personnic

Last updated by author(s): Aug 22, 2019

Reporting Summary

Nature Research wishes to improve the reproducibility of the work that we publish. This form provides structure for consistency and transparency in reporting. For further information on Nature Research policies, see Authors & Referees and the Editorial Policy Checklist.

Statistics

For all statistical analyses, confirm that the following items are present in the figure legend, table legend, main text, or Methods section.

n/a	Confirmed	
☐	☒	The exact sample size (n) for each experimental group/condition, given as a discrete number and unit of measurement
☐	☒	A statement on whether measurements were taken from distinct samples or whether the same sample was measured repeatedly
☐	☒	The statistical test(s) used AND whether they are one- or two-sided<br>*Only common tests should be described solely by name; describe more complex techniques in the Methods section.*
☐	☒	A description of all covariates tested
☐	☒	A description of any assumptions or corrections, such as tests of normality and adjustment for multiple comparisons
☐	☒	A full description of the statistical parameters including central tendency (e.g. means) or other basic estimates (e.g. regression coefficient) AND variation (e.g. standard deviation) or associated estimates of uncertainty (e.g. confidence intervals)
☐	☒	For null hypothesis testing, the test statistic (e.g. F, t, r) with confidence intervals, effect sizes, degrees of freedom and P value noted<br>*Give P values as exact values whenever suitable.*
☒	☐	For Bayesian analysis, information on the choice of priors and Markov chain Monte Carlo settings
☒	☐	For hierarchical and complex designs, identification of the appropriate level for tests and full reporting of outcomes
☒	☐	Estimates of effect sizes (e.g. Cohen's d, Pearson's r), indicating how they were calculated

Our web collection on statistics for biologists contains articles on many of the points above.

Software and code

Policy information about availability of computer code

Data collection	Data collection is described in detail in the methods section and using the following devices:<br>Flow-cytometry analyser FACS-Fortessa II<br>Image flow cytometry: ImageStream X MkII imaging flow cytometer<br>Sorter: Aria II<br>Confocal microscopy: Leica SP8
Data analysis	Flow-cytometry/Sorting: FlowJo<br>Image flow cytometry: IDEAS 6.2<br>Confocal microscopy: ImageJ/LasX

For manuscripts utilizing custom algorithms or software that are central to the research but not yet described in published literature, software must be made available to editors/reviewers. We strongly encourage code deposition in a community repository (e.g. GitHub). See the Nature Research guidelines for submitting code & software for further information.

Data

Policy information about availability of data

All manuscripts must include a data availability statement. This statement should provide the following information, where applicable:

- Accession codes, unique identifiers, or web links for publicly available datasets
- A list of figures that have associated raw data
- A description of any restrictions on data availability

All data is available in the main text or the supplementary material and provided as source data files. The mass spectrometry proteomics data have been deposited to the ProteomeXchange Consortium via the PRIDE partner repository with the dataset identifier PXD015106 [http://proteomecentral.proteomexchange.org/cgi/GetDataset?ID=PXD015106].

All other relevant data and mMaterial will be provided upon request to npersonnic@imm.uzh.ch

Field-specific reporting

Please select the one below that is the best fit for your research. If you are not sure, read the appropriate sections before making your selection.

☒ Life sciences ☐ Behavioural & social sciences ☐ Ecological, evolutionary & environmental sciences

For a reference copy of the document with all sections, see nature.com/documents/nr-reporting-summary-flat.pdf

Life sciences study design

All studies must disclose on these points even when the disclosure is negative.

Sample size	For Flow cytometry analysis at least 20'000 events per tested sample<br>For Sorting: a minimum of 100'000 events per tested sample<br>For Image floy cytometry: a minimum of 20'000 events per tested sample<br>For microscopy: a minimum of 50 events per tested samples
Data exclusions	We did not exclude any data
Replication	Number of repat are indicated on the figure legends. Experiments were analyzed using a minimum of three independent biological replicates (highlighted by light grey filled circle on the bar charts).
Randomization	Samples were allocated based on the genetical as well as phenotypical background.
Blinding	We are mostly comparing subpopulations x1 and x2 within the clonal population X. Our work is based on the combination of high-throughput techniques with fluorescent based reporters preventing from biaised analysis. Samples involving various genetical or phenotypical bacgrounds were blinded prior to analysis.

Reporting for specific materials, systems and methods

We require information from authors about some types of materials, experimental systems and methods used in many studies. Here, indicate whether each material, system or method listed is relevant to your study. If you are not sure if a list item applies to your research, read the appropriate section before selecting a response.

Materials & experimental systems

n/a	Involved in the study	
☐	☐	Antibodies
☐	☒	Eukaryotic cell lines
☐	☐	Palaeontology
☐	☐	Animals and other organisms
☐	☐	Human research participants
☐	☐	Clinical data

Methods

n/a	Involved in the study	
☐	☐	ChIP-seq
☐	☒	Flow cytometry
☐	☐	MRI-based neuroimaging

Antibodies

Antibodies used	do not apply
Validation	do not apply

Eukaryotic cell lines

Policy information about cell lines

Cell line source(s)	Acanthamoeba castellanii (ATCC 30234) / Dictyostelium discoideum wild-type strain Ax3 (Loovers, H.M. et al. 2007) / Maf-DKO murine macrophages (Aziz A. et al. 2009)
Authentication	Cell lines were obtained from ATCC or from the lab that first published them.
Mycoplasma contamination	Cell lines negative to mycoplasma
Commonly misidentified lines (See ICLAC register)	do not apply

Palaeontology

Specimen provenance	do not apply
Specimen deposition	do not apply
Dating methods	do not apply

☐ Tick this box to confirm that the raw and calibrated dates are available in the paper or in Supplementary Information.

Animals and other organisms

Policy information about studies involving animals; ARRIVE guidelines recommended for reporting animal research

Laboratory animals	do not apply
Wild animals	do not apply
Field-collected samples	do not apply
Ethics oversight	do not apply

Note that full information on the approval of the study protocol must also be provided in the manuscript.

Human research participants

Policy information about studies involving human research participants

Population characteristics	do not apply
Recruitment	do not apply
Ethics oversight	do not apply

Note that full information on the approval of the study protocol must also be provided in the manuscript.

Clinical data

Policy information about clinical studies

All manuscripts should comply with the ICMJE guidelines for publication of clinical research and a completed CONSORT checklist must be included with all submissions.

Clinical trial registration	do not apply
Study protocol	do not apply
Data collection	do not apply
Outcomes	do not apply

ChIP-seq

Data deposition

☐ Confirm that both raw and final processed data have been deposited in a public database such as GEO.

☐ Confirm that you have deposited or provided access to graph files (e.g. BED files) for the called peaks.

Data access links<br>*May remain private before publication.*	do not apply
Files in database submission	do not apply
Genome browser session<br>(e.g. UCSC)	do not apply

Methodology

Replicates	do not apply

Sequencing depth	do not apply
Antibodies	do not apply
Peak calling parameters	do not apply
Data quality	do not apply
Software	do not apply

Flow Cytometry

Plots

Confirm that:

☒ The axis labels state the marker and fluorochrome used (e.g. CD4-FITC).

☒ The axis scales are clearly visible. Include numbers along axes only for bottom left plot of group (a 'group' is an analysis of identical markers).

☒ All plots are contour plots with outliers or pseudocolor plots.

☒ A numerical value for number of cells or percentage (with statistics) is provided.

Methodology

Sample preparation	The sample preparation is detailed in the methods section.<br>Flow-cytometry: Phagocytes were infected with L. pneumophila wild-type or the isogenic deletion mutant strains and lysed using 0.1% Triton TX-100 (Sigma) in 150 mM NaCl (amoebae) or PBS (macrophages). After centrifugation the pellets were washed in PBS and fixed with 2% paraformaldehyde / 0.1% glutaraldehyde for 60 min. Following fixation cells were washed and the fixative was quenched with 0.1 M glycine for 20min at room temperature. Relevant spectral parameters were subsequently recorded in a FACS-Fortessa II<br><br>Image flow cytometry: The cells were detached and fixed with 2% paraformaldehyde (Electron Microscopy Sciences) for 60 min, and the fixative was quenched with 0.1M glycine for 20min at room temperature. At least 20’000 events were acquired using an ImageStream X MkII imaging flow cytometer (Amnis). Data analysis was performed with IDEAS 6.2 software, and after color compensation.<br><br>Sorting: A. castellanii infected for 24 h with timer expressing L. pneumophila were lysed using 0.1% Triton TX-100 (Sigma) in HS buffer (20 mM N -2-hydroxyethylpiperazine-N-2-ethanesulfonic acid (HEPES); 250 mM sucrose; 0.5 mM ethyleneglycoltetraacetic acid (EGTA); pH adjusted to 7.2 with 1 M KOH). Lysates were centrifuged (250 g, 15 min), and resuspended in PBS, followed by nine passages through a ball homogenizer (Isobiotech, http://www.isobiotec.com) using an exclusion size of 6 um. Samples were sorted according to Relevant spectral parameters using an Aria II (BD Biosciences) with scatter and fluorescence channels, a nozzle size of 70 um using the 4-way purity mode and a sorting efficiency > 90%. Sorted subsets were systematically reanalyzed for purity assessment.
Instrument	Flow-cytometry analyser: FACS-Fortessa II<br>Image flow cytometry: ImageStream X MkII imaging flow cytometer<br>Sorter: Aria II
Software	FACSDiva for collection<br>Flow-cytometry/Sorting: FlowJo<br>Image flow cytometry: IDEAS 6.2
Cell population abundance	As previoulsy stated, the population of interest was:<br>For Flow cytometry analysis at least 20'000 events per tested sample<br>For Sorting: a minimum of 100'000 events per tested sample<br>For Image flow cytometry: a minimum of 20'000 events per tested sample
Gating strategy	The gating strategy is depicted in the supplemental fig. 2c and 2d. Flow-cytometry analysis of fluorescent L. pneumophila in infected cell lysates: Cell were infected with L. pneumophila producing a fluorescent protein and lysed to release intracellular bacteria prior to flow-cytometry analysis. FSC-H vs SSC-H was used to descriminate intact cells from released bacteria (gate 1). Then, the spectral properties of the fluorescent protein allow to separate the signals from host autofluorescence (gate 2/within gate 1). The use of non-fluorescent L. pneumophila confirms the absence of any background particles with similar fluorescence. gate 3 and gate 4 (within gate 2) correspond to the subpopulations expressing alternative phenotype based on the fluorescent reporter used. Gate 2 and 3 are used for subsequent sorting experiments.

☒ Tick this box to confirm that a figure exemplifying the gating strategy is provided in the Supplementary Information.

Magnetic resonance imaging

Experimental design

Design type	do not apply
Design specifications	do not apply
Behavioral performance measures	do not apply

Acquisition

Imaging type(s)	do not apply
Field strength	do not apply
Sequence & imaging parameters	do not apply
Area of acquisition	*State whether a whole brain scan was used OR define the area of acquisition, describing how the region was determined.*

Diffusion MRI ☐ Used ☒ Not used

Preprocessing

Preprocessing software	do not apply
Normalization	do not apply
Normalization template	do not apply
Noise and artifact removal	do not apply
Volume censoring	do not apply

Statistical modeling & inference

Model type and settings	do not apply
Effect(s) tested	do not apply

Specify type of analysis: ☐ Whole brain ☐ ROI-based ☐ Both

Statistic type for inference (See Eklund et al. 2016)	do not apply
Correction	do not apply

Models & analysis

n/a	Involved in the study
☒	☐ Functional and/or effective connectivity
☒	☐ Graph analysis
☒	☐ Multivariate modeling or predictive analysis
